# 

**Published:** 1899-04

**Authors:** 


### CONTENTS.

ORIGINAL COMMUNICATIONS—
FIBROID TUMORS OF THE UTERUS. By Virgil O. Hardon, M.D , Atlanta, Ga..........
PROTECTION AGAINST FRAUDULENT MALPRACTICE SUITS. By Bernard Wolff, M.D., Atlanta, Ga..........
CIRCUMCISION. By Thomas H. Hancock, M.D., Atlanta, Ga..........
THE USE AND ABUSE OF THE X-RAY IN SURGERY. By J. McF. Gaston, Jr., A.M., M.D., Atlanta, Ga..........
MULTIPLE NEURITIS FROM ARSENICAL POISONING. By Isham Harrison, M.D., Deerbro[illegible]k, Miss..........
A FEW WORDS IN REFERENCE TO SOME MEDICAL USES OF FORMALDEHYDE. By W. H. Hudson, M.D., Lafayette, Ala..........

SOCIETY REPORTS—
ATLANTA SOCIETY OF MEDICINE..........
PROCEEDINGS OF THE MEMPHIS MEDICAL SOCIETY..........

CORRESPONDENCE—
AMERICAN MEDICAL ASSOCIATION—ANNUAL ANNOUNCEMENT..........

EDITORIAL NOTES AND COMMENTS—
ANNOUNCEMENT..........
THE ATLANTA COLLEGE OF PHYSICIANS AND SURGEONS..........
THE CODDLING OF CHILDREN TO PREVENT COLDS..........
DISEASE GERMS AND THE TELEPHONE..........

NEWS AND NOTES..........

MEDICAL MUSINGS..........

BOOK REVIEWS, PAMPHLETS, EXCHANGES—
BOOK REVIEWS..........
REPRINTS RECEIVED..........

[illegible]EDICAL ITEMS..........xii, xiii, xiv, xvi, xvii, xviii, xx, [illegible]

[illegible]VERTISERS' INDEX TO ADVERTISEMENTS..........

